# Strong synergy with APR-246 and DNA-damaging drugs in primary cancer cells from patients with *TP53* mutant High-Grade Serous ovarian cancer

**DOI:** 10.1186/s13048-016-0239-6

**Published:** 2016-05-14

**Authors:** Åsa Fransson, Daria Glaessgen, Jessica Alfredsson, Klas G. Wiman, Svetlana Bajalica-Lagercrantz, Nina Mohell

**Affiliations:** Aprea AB, Solna, Sweden; Karolinska University Hospital, Stockholm, Sweden; Karolinska Institutet Dept. of Oncology-Pathology, Cancer Center Karolinska (CCK), Stockholm, Sweden

**Keywords:** Ovarian cancer, High-Grade Serous (HGS) cancer, APR-246 (PRIMA-1^MET^), Primary cancer cells, *TP53* mutation, p53 reactivation, DNA-damaging drugs, Cisplatin, Doxorubicin, Synergy

## Abstract

**Background:**

Mutation in the tumor suppressor gene *TP53* is an early event in the development of high-grade serous (HGS) ovarian cancer and is identified in more than 96 % of HGS cancer patients. APR-246 (PRIMA-1^MET^) is the first clinical-stage compound that reactivates mutant p53 protein by refolding it to wild type conformation, thus inducing apoptosis. APR-246 has been tested as monotherapy in a Phase I/IIa clinical study in hematological malignancies and prostate cancer with promising results, and a Phase Ib/II study in combination with platinum-based therapy in ovarian cancer is ongoing. In the present study, we investigated the anticancer effects of APR-246 in combination with conventional chemotherapy in primary cancer cells isolated from ascitic fluid from 10 ovarian, fallopian tube, or peritoneal cancer patients, 8 of which had HGS cancer.

**Methods:**

Cell viability was assessed with fluorometric microculture cytotoxicity assay (FMCA) and Combination Index was calculated using the Additive model. p53 status was determined by Sanger sequencing and single strand conformation analysis, and p53 protein expression by western blotting*.*

**Results:**

We observed strong synergy with APR-246 and cisplatin in all tumor samples carrying a *TP53* missense mutation, while synergistic or additive effects were found in cells with wild type or *TP53* nonsense mutations. Strong synergy was also observed with carboplatin or doxorubicin. Moreover, APR-246 sensitized *TP53* mutant primary ovarian cancer cells, isolated from a clinically platinum-resistant patient, to cisplatin; the IC_50_ value of cisplatin decreased 3.6 fold from 6.5 to 1.8 μM in the presence of clinically relevant concentration of APR-246.

**Conclusion:**

These results suggest that combination treatment with APR-246 and DNA-damaging drugs could significantly improve the treatment of patients with *TP53* mutant HGS cancer, and thus provide strong support for the ongoing clinical study with APR-246 in combination with carboplatin and pegylated liposomal doxorubicin in patients with recurrent HGS cancer.

## Background

Ovarian, fallopian tube, and peritoneal high-grade serous (HGS) cancers share many characteristics and are clinically managed in the same way [1]. The cancer is classified based on characteristics like main tumor burden and immunohistological pattern. Ovarian cancer is the most common tumor in this group. Accumulated evidence suggests that HGS cancer found in these tissues shares a similar origin and pathogenesis, pointing out the fallopian tube as the origin in the majority of cases [2, 3]. HGS cancer is the most malignant form of ovarian cancer and accounts for 70–80 % of deaths [4]. Despite intense research to find novel treatments there has been minimal improvement in overall survival of the patients for the past decades [4]. Although most HGS patients respond well to first-line treatment with platinum-based therapy, acquired resistance to conventional cancer therapies and high death rates remain a major challenge. Thus, novel treatment strategies directed against cancer-specific targets are urgently needed.

Mutation in the *TP53* gene, which encodes the tumor suppressor protein 53 (p53), is an early event in the development of HGS cancer. More than 96 % of patients with HGS cancer have mutations in *TP53*, which supports the idea that mutations in *TP53* are driver mutations essential for the early development of this disease [5]. *TP53* mutations are common also in other types of ovarian cancer and occur in at least 60 % of all ovarian tumors [6]. p53 induces cell cycle arrest, senescence and/or apoptosis upon various types of cellular stress, such as DNA damage, oncogene activation, and hypoxia. Mutations in *TP53* are identified in about 50 % of all tumors and are associated with increased resistance to chemotherapy and reduced survival in many tumor types [7, 8].

APR-246 is the first clinical phase small molecule that has been shown to reactivate mutant or otherwise incorrectly folded p53-protein by promoting its active conformation [9, 10]. It is a prodrug that is converted to the active form MQ (2-methylenequinuclidin-3-one), a Michael acceptor that binds to mutant p53 and restores the active wild type conformation, thus triggering apoptosis [9, 10]. APR-246 has been tested as monotherapy in a Phase I/IIa clinical trial in patients with hematological malignancies and prostate cancer, with encouraging results [11]. A Phase Ib/II Proof of Concept study with APR-246 in combination with carboplatin and pegylated liposomal doxorubicin is ongoing in patients with recurrent *TP53*-mutant HGS cancer.

We have previously demonstrated strong synergistic anticancer effects with APR-246 and DNA-damaging drugs in ovarian cancer cell lines with various p53 status [12]. Moreover, we and others have shown that APR-246 (or the analog PRIMA-1) can sensitize p53 mutant chemoresistant cancer cell lines to cisplatin [12, 13]. However, cell lines often show substantial differences from original tumors. Indeed, pronounced differences between some of the most commonly used HGS ovarian cancer cell line models and the majority of HGS ovarian cancer samples have been revealed [14, 15]. The aim of this study was to investigate the anticancer effect of APR-246 as single compound as well as in combination with conventional chemotherapeutic drugs, platinum compounds and doxorubicin, in primary cancer cells isolated from ascites (i.e., accumulated fluid in the abdominal cavity) from ovarian, fallopian tube, or peritoneal cancer patients.

## Methods

### Test substances

- APR-246, also called PRIMA-1MET (2-hydroxymethyl-2-methoxymethyl-1-azabicyclo [2, 2, 2] octan-3-one) (Batch No. GF707504, Syngene)
- Cisplatin (Product No. 020345, Ebewe or Product No. 146262, Hospira)
- Carboplatin (Product No. 136164, Hospira)
- Doxorubicin (Product No. 021361, Teva)

### Primary cancer cells

Cancer cells isolated from ascitic fluid of 10 evaluable patients were included in the study. Written informed consent was obtained before ascitic fluid was collected, and the procedures were in agreement with the Swedish law and ethical regulations. The research project was approved by the local ethics committee at Karolinska Institutet (Dnr 2012/1933-31), including scientific publishing, and is in accordance with the principles of the Declaration of Helsinki. All samples were from patients with serous adenocarcinoma and 8 of them fulfilled the present criteria of high-grade serous type (Table 1) [16]. According to the recommendations described in the Swedish National Guidelines for Ovarian Cancer, for all patients diagnosed after January 1^st^, 2014, the histopathological grading of ovarian serous carcinoma was performed using a two-tier system [17]. For the patients included in the study, and diagnosed before 2014, the histological slides were not reevaluated. Nevertheless, the pathological report was matched for the present criteria. Specific information about diagnosis, histology, treatment, and p53 status is listed in Table 1. 8 samples were from patients who had previously received chemotherapy.

**Table 1:** Summary of results with APR-246 and cisplatin in primary cancer cells from the patients

Ascites sample	Diagnosis	Histological description	Prior chemo-therapy	Platinum sensitivity	IC_50_ APR-246 (μM)	IC_50_ cisplatin (μM)	p53 status	p53 protein levels	Combination APR-246 cisplatin
1	Ovarian cancer stage III	Poorly differentiated serous carcinoma^a^	Yes	Resistant	25	40^*^	R280K (hom.)	++	SS
2	Cancer peritonei stage IV	Serous carcinoma	Yes	Resistant	24	18	wt	−	S, Add
3	Ovarian cancer stage IC	Serous carcinoma	Yes	Resistant	37	32	L111Q (het.)	+	S, SS
4	Ovarian cancer/Cancer peritonei stage IV	Poorly differentiated serous carcinoma^a^	Yes	Resistant	18	3.2	P151H (het.)	+	SS
5	Cancer peritonei stage IIIC	Serous adenocarcinoma, grade III^a^	No	Sensitive	22	16	C135Y (hom.)	+	SS
6	Tubar cancer stage IIIB	Poorly differentiated serous adenocarcinoma^a^	Yes	Sensitive	12	17	C238F (het.)	++	SS
7	Ovarian cancer stage IIIC	Poorly differentiated serous carcinoma^a^	Yes	Resistant	56^d^	45^d^	E346^*^ (het.)	−	S, Add
8	Cancer Peritonei stage IV	Poorly differentiated serous carcinoma^a^	No	Sensitive	8.9	7.1	E204^*^ (hom.)	−	SS
9	Ovarian cancer stage IIIC	Poorly differentiated serous carcinoma^a^	Yes	Resistant	5.2	7.9	P278R (hom.)	+	SS
10	Ovarian cancer stage IIIC	Medium-well differentiated serous adenocarcinoma^a,b^	Yes	Sensitive	8.0	17	Y163H (het.)	+	SS

Cell viability was assessed by the FMCA assay. Combination Index (CI) was calculated using the Additive model.

*Diagnosis:* The 10 samples included in the study were from patients diagnosed with ovarian, peritoneal, or fallopian tube cancer. Strong data suggest that high-grade serous (HGS) carcinomas found in these tissues share a similar origin and pathogenesis pointing out the distal part of the fallopian tubes as the origin in the majority of cases [2, 3].

*Histological description:* The primary histopathological analysis of the samples in this study was performed using the previous grading system and heterogeneous criteria and not according to the present standard where tumors are classified into low- and high-grade serous carcinomas.

^a^These samples are classified as HGS carcinoma according to the current criteria [16]. Likely is also sample 3 with *TP53* mutation HGS carcinoma.

^b^Patient 10 was later reoperated for distant metastasis and the histopathological analysis showed HGS tumor.

*Platinum sensitivity:* patients who relapse > 6 months are classified as platinum-sensitive; patient who relapse < 6 months are platinum-resistant.

*p53 status:* het. = heterozygous; hom. = homozygous; ^*^ = stop codon. The sequencing method used cannot distinguish homozygous from hemizygous mutations, neither can heterozygosity be distinguished from a mixture of cells with wild type and mutant *TP53*.

*p53 protein levels:*− = not detected; + = medium; ++ = high.

*Combination APR-246 cisplatin:* Add = additive effect (CI = 1.0 ± 0.2); S = synergy (CI < 0.8); SS = strong synergy (CI < 0.5).

^c^The dose-response curve of cisplatin levelled off and thus resulted in difficulty to determine the IC_50_ value.

^d^Cisplatin and APR-246 had high IC_50_ values in sample 7. Moreover, results from combination experiments in this sample were variable resulting in high standard error.

Ascites was drained by abdominal paracenthesis from patients with ovarian, fallopian tube, or peritoneal cancer as symptomatic treatment. The procedure resulted in 0.5–6 l of ascitic fluid/patient. Heparin (LEO 5000 IE/Ky/ml, Lot: 015227-06/DG2741, Leo Pharma) was added to each bag (5 ml/l ascites) prior to the ascites drainage. 0.5-3 l ascitic fluid was centrifuged in 50 ml Falcon tubes at 200 g for 5 min (room temperature) and each cell pellet was resuspended in 30 ml CO_2_-independent medium (#18045-054, Gibco), supplemented with 5 % heat inactivated fetal bovine serum (FBS) (#F7524, Sigma), 1 mM L-glutamine (#7513, Sigma), and 5000u streptomycin and 5 mg penicillin/ml (#P0781, Sigma). To the bottom of each tube, 10 ml Ficoll paque plus (Batch No. 17-1440-02, GE Healthcare) was added with a syringe. The tubes were centrifuged at 510 g for 15 min. The tumor cell-rich fraction, in the interface between the medium fraction and the Ficoll fraction, was removed with a Pasteur pipette and transferred to a fresh tube. The cells were washed three times in supplemented CO_2_-independent medium and counted in Bürker’s chamber. Most of the samples had cell aggregates of various sizes which resulted in difficulties in determining exact cell count. Samples 3 and 6 with large aggregates were filtered through 100 μm filter. Cytospin glasses were prepared (~10000 cells/well). Thereafter, the remaining cells were viably frozen in 90 % FBS + 10 % DMSO.

### Assessment of cell viability

Cell viability was assessed by the fluorometric microculture cytotoxicity assay (FMCA), which measures the esterase activity of cells with intact plasma membrane by hydrolysis of the nonfluorescent probe fluorescein diacetate (FDA) to fluorescein [18]. The generated fluorescence is proportional to the number of cells with intact plasma membrane, i.e., viable cells.

96-well plates with v-shaped wells (# 249570, NUNC) were prepared with test substances at 10x the desired concentration. The substance plates were sealed with microtiter tape and kept at -80 °C until used. The cell suspension was seeded (10000 cells/well in a total volume of 200 μl) in thawed substance plates and incubated for 72 h at 37 °C with 5 % CO_2_. After incubation, the cell plates were washed and incubated with 100 μl per well of 1 μl FDA (# F7278, Sigma)/ml physiological buffer (Q2) [18] for 40 min at 37 °C with 5 % CO_2_. Thereafter, the fluorescence generated (excitation 480 nm) was measured at 538 nm.

### Analysis of synergy

Combination effects were analyzed using the Additive model [19–21]. The model assumes a log-linear shape of the dose-response curve, which is fairly valid if the drug concentrations used cause effects in the steep part of the dose-response curve [21]. In samples with two co-incubated substances, a predicted cell viability (%) is calculated according to the following formula: Predicted cell viability (%) = cell viability of substance 1 (%) x cell viability of substance 2 (%) x 0.01. Combination Index (CI) is then calculated as the measured cell viability of the sample with two co-incubated substances divided by the predicted cell viability. CI 0.8–1.2 is considered as an additive effect (the interval of 1.0 ± 0.2 is set to account for intra-assay variability). CI < 0.8 is synergistic effect and CI > 1.2 sub-additive effect. If the measured cell viability for a combination of two substances is higher than the cell viability for one or both of the substances, the effect is considered antagonistic. In this report, we have classified CI < 0.5 as strong synergistic effect. If the predicted viability is very low, the quote “measured viability/predicted viability” may give false CI values. Thus, we decided to set a lower limit of 5 % for the predicted viability.

### *TP53* sequencing and western blotting

For all samples used in the study, the *TP53* gene was analyzed by Sanger sequencing and single strand conformation analysis and the p53 protein expression by western blotting. The *TP53* gene status (exons 2–11) was analyzed by PCR amplification and sequencing, followed by single-strand conformation analysis (SSCA) according to the original protocol [22], and samples displaying gel mobility shifts were sequenced to confirm the nucleotide change.

Western blotting: Cells were lysed in RIPA buffer (2x RIPA: 0,87766 g NaCl, 1 ml 100x Triton, 0.5 ml 20 % SDS, 5 ml 1 M Tris pH 8.0 + H_2_O up to 50 ml) supplemented with protease inhibitor (#04906845001, Roche) for 30 min on ice. After centrifugation the supernatant was isolated and used for further analysis. Total protein concentration was determined with the BCA-method. Equal amount of protein from all samples was added to 2x Laemmli buffer (#161-0737, BioRad) supplemented with 2-mercaptoethanol (#161-0710, BioRad, 1:20 in the Laemmli buffer) in a 1:1 ratio, and boiled at 95 °C for 5 min before separated by electrophoresis on a 4–15 % TGX-gel (BioRad) and transferred to PVDF membrane using a semi-dry trans blot system (BioRad). The membranes were blocked in Tris-buffered saline containing 5 % *w/v* nonfat dry milk and 0.1 % Tween-20, followed by incubation with primary antibody overnight at 4 °C, and with secondary antibody for 1 h at room temperature.

Primary antibodies: anti-p53 antibody (#9282, Cell Signaling), anti-p53 antibody (#FL-393, Santa Cruz), anti-GAPDH antibody (#5632-1, clone EPR6256, Epitomics). Secondary antibodies: HRP-conjugated antibodies (#P 0447 or #P 044801-2, Dako). The proteins were visualized by enhanced chemiluminescence system (ECL prime, #RPN2232, VWR) and detected using a CCD camera (LAS1000 Fujifilm Tokyo Japan). Dark pixels were measured for quantification of protein bands using ImageJ.

*TP53* sequencing and western blotting were performed at the Department of Clinical and Experimental Medicine, Linköping University (Dr. Peter Söderkvist).

### Microscopic analysis of cells

May Grünwald-Giemsa stained cytospin slides were visually judged by an experienced cytopathologist (Manuel de la Torre, Uppsala University Hospital). According to our quality criteria, at least 70 % of all cells in a sample should be cancer cells and the cell viability should be at least 70 %. All samples included in the study met these criteria. For some samples immunostainings with anti-BER-EP4 antibody (# IR637, Dako) and anti-calretinin antibody (clone NCL-L-CALRETININ, Novacasta) were needed in order to discriminate between cancer cells and mesothelial cells. The anti-BER-EP4 antibody stains all cells of epithelial origin including ovarian cancer cells, whereas the anti-calretinin antibody stains mesothelial cells. Secondary staining was performed with Dako REAL EnVision Detection System (#K5007) and visualized with diaminobenzidine (DAB) according to the kit.

## Results

### Characteristics of the patients

In this study, primary cancer cells isolated from ascitic fluid from 10 patients were investigated. 8 patients had previously received platinum-based chemotherapy. The patients were diagnosed with ovarian, peritoneal, or fallopian tube cancer and 8 of them fulfilled the current histopathological criteria for HGS cancer (Table 1).

### p53 status

**Fig. 1:**
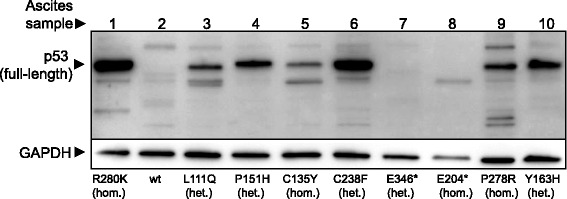
Expression of p53 in primary ovarian cancer cells. The anti-p53 antibody used (polyclonal antibody #9282) binds strongly to the N-terminus and weakly to the DNA-binding region of p53. * = stop codon, hom. = homozygous. het. = heterozygous. It should be noted that the sequencing method used cannot distinguish between homozygous and hemizygous mutations. “het.” indicates that both wild type p53 and mutant p53 are found in the sample, which can either be due to heterozygosity or heterogeneity in the sample with the presence of cells with different p53 status

As shown in Fig. 1, cancer cells from 7 patients possessed *TP53* missense mutations leading to the following substitutions in the DNA-binding domain of p53: L111Q, C135Y, P151H, Y163H, C238F, P278R, and R280K, respectively. Two patients had nonsense mutations, and one had wild type p53. p53 proteins accumulated at medium or high levels in cells with missense mutations, while no p53 protein was detected with anti-p53 antibody (#9282) in cells carrying wild type or nonsense mutant *TP53* (Fig. 1, Table 1). Similar results were obtained also with another anti-p53 antibody (FL-393, data not shown).

### Sensitivity of primary ovarian cancer cells to APR-246 and conventional drugs

**Fig. 2:**
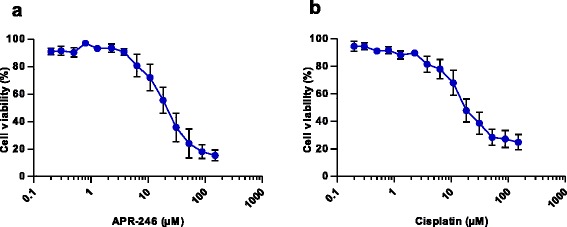
Inhibition of cell viability by APR-246 (**a**) and cisplatin (**b**). Cell viability was assessed by the FMCA assay after 72 h incubation with drug. The results shown are mean ± SEM; *n* = 10 patients

The sensitivity of primary ovarian cancer cells to APR-246 and DNA-damaging drugs was investigated using the FMCA cell viability assay. Fig. 2 shows that both APR-246 and cisplatin decreased cell viability in a dose-dependent manner. Table 1 summarizes the individual IC_50_ values of APR-246 and the platinum drug cisplatin. The mean IC_50_ value of APR-246 was 22 ± 4.9 μM and that of cisplatin 20 ± 4.5 μM (mean ± SEM; *n* = 10 patients).

The sensitivity of the cisplatin analog carboplatin and the anthracycline doxorubicin was also investigated. The mean IC_50_ values of carboplatin and doxorubicin were 47 ± 15 μM and 5.9 ± 2.1 μM, respectively (mean ± SEM; *n* = 5 patients). The individual IC_50_ values of carboplatin ranged from 3.7 to 87 μM and those of doxorubicin from 1.2 to 12 μM (data not shown).

### Strong synergistic effects with APR-246 and DNA-damaging drugs in primary ovarian cancer cells carrying missense *TP53* mutations

**Fig. 3:**
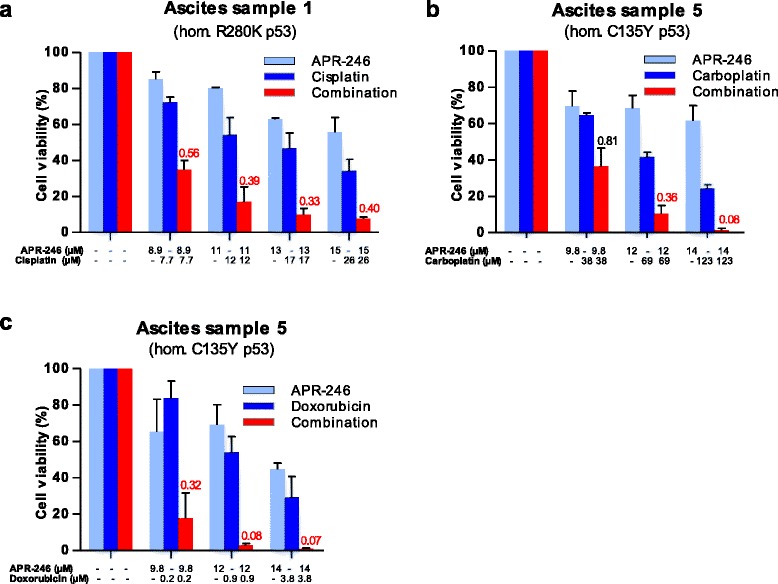
Strong synergy with APR-246 and cisplatin (**a**), carboplatin (**b**), or doxorubicin (**c**) in primary ovarian cancer cells. Cell viability was assessed by FMCA assay after 72 h incubation with drugs. Additive model was used for analysis of combination effects. Combination Index (CI) values are presented above the bars. CI < 0.8 indicates synergy and < 0.5 strong synergy. CI values < 0.8 are marked in red. The results shown are mean ± SEM (*n* = 2)

We then investigated combination effects of APR-246 and cisplatin in cancer cells with various p53 status. As shown in Fig. 3a, strong synergistic effect (CI < 0.5) was observed in cancer cells carrying homozygous R280K p53. Table 1 summarizes the results from studies using cancer cells from all 10 patients. Strong synergy with APR-246 and cisplatin was found in all cancer cells carrying *TP53* missense mutations. In sample 3, synergistic effects (CI < 0.8) were also observed. Additive or synergistic effects were found in cancer cells carrying wild type *TP53* or nonsense *TP53* mutation in the tetramerization domain resulting in truncated E346* p53. Strong synergy was observed in cells carrying the homozygous nonsense *TP53* mutation resulting in truncated E204* p53.

Combination studies with APR-246 and carboplatin (Fig. 3b) or doxorubicin (Fig. 3c) were performed in 2 samples carrying homozygous *TP53* missense mutation (sample 5 and 9). Strong synergistic effects with APR-246 in combination with carboplatin or doxorubicin were obtained in cancer cells from both samples.

### APR-246 sensitizes primary ovarian cancer cells to cisplatin

**Fig. 4:**
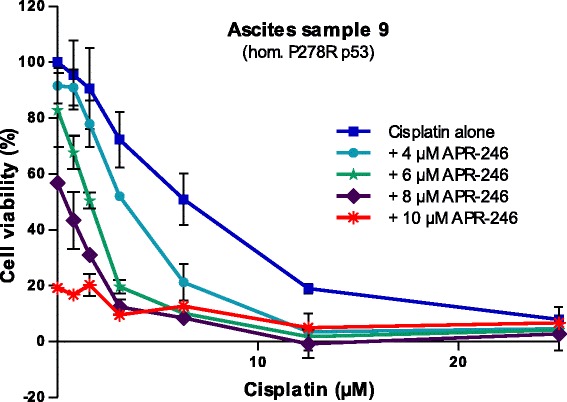
APR-246 sensitized primary ovarian cancer cells with *TP53* mutation to cisplatin. The FMCA assay was used to assess cell viability after 72 h incubation with drugs. This ascites sample was taken from a clinically platinum-resistant patient. The results shown are mean ± SEM (*n* = 2)

We also tested the ability of APR-246 to sensitize primary cancer cells to cisplatin, using cells from a recurrent clinically platinum-resistant patient carrying homozygous P278R mutant *TP53*. Dose-response experiments with cisplatin alone and in combination with various concentration of APR-246 were performed. As shown in Fig. 4, APR-246 sensitized the primary cancer cells to cisplatin in a dose-dependent manner; the IC_50_ value of cisplatin (with the partial effect contribution from APR-246 subtracted) decreased 3.6-fold from 6.5 to 1.8 μM in the presence of 8 μM APR-246.

## Discussion

Most HGS tumors are detected at an advanced stage since symptoms are usually not apparent until the cancer has metastasized. The standard treatment of advanced ovarian cancer is cytoreductive surgery followed by 6-8 cycles of platinum compounds in combination with taxanes [23]. Many patients respond well to the first-line treatment but most of the patients relapse. Second-line therapy can prolong survival but is rarely curative. For platinum-sensitive patients (i.e., those who relapse > 6 months) treatment with carboplatin, in combination with paclitaxel, pegylated liposomal doxorubicin, or gemcitabine, is the standard therapy [24]. For patients with platinum-resistant disease (who relapse < 6 months) single agent therapy with, e.g., pegylated liposomal doxorubicin, is the standard treatment.

Platinum-based drugs have for decades been used for effective treatment of many solid tumors, including ovarian cancer [25, 26]. Cisplatin, the first member of this class, has had a major impact on the treatment of cancer but is also associated with side effects, including nephrotoxicity. This led to the development of the less toxic analog carboplatin [25]. Although platinum compounds exert their anticancer effect by multiple mechanisms, their primary mechanism of action is interaction with DNA to form DNA-adducts, which leads to DNA damage response and p53-dependent apoptosis. However, repeated treatment with platinum compounds leads to a gradual decrease of the DNA damage response and resistance to platinum drugs as well as cross-resistance to other DNA-damaging drugs. The underlying mechanism of platinum resistance is multifactorial and involves drug-induced increase in intracellular glutathione (GSH) levels leading to enhanced efflux of platinum compounds, reduced drug uptake, increased drug inactivation, as well as inactivation of the tumor suppressor protein p53 [26–29].

The mode of action of APR-246 as a p53-mutant targeting compound is well documented, and the molecular mechanisms underlying the reactivation of mutant p53 and induction of apoptosis are relatively well characterized [9, 10, 30]. APR-246 is a prodrug that is converted to MQ, a Michael acceptor that binds to cysteine residues in mutant and/or unfolded p53 and promotes correct folding. This induces activation of caspase-2 and expression of p53-target genes, including, *PUMA*, *NOXA,* and *BAX,* leading to mitochondrial apoptosis [9, 10, 30]. Recently, we have shown that MQ also binds to cysteine residues in glutathione and thereby decreases intracellular free glutathione concentrations in drug-resistant *TP53*-mutant ovarian cancer cells [12]. Moreover, APR-246 can bind to free cysteines leading to inhibition of glutathione synthesis and to further decrease in intracellular glutathione concentration. APR-246 can also trigger apoptosis in a p53-independent manner by binding to thioredoxin reductase 1 (TrxR1) and inducing ROS [9, 10, 12, 31]. This unique mechanism of action of APR-246, targeting both p53 and glutathione, probably underlies the outstanding synergistic effect as well as the complete resensitization to cisplatin observed with APR-246 in combination with cisplatin in drug-resistant *TP53* mutant ovarian cancer cell lines [12].

In the present study we have investigated the effects of APR-246 in combination with current therapy in primary ovarian cancer cells isolated from ascitic fluid. Cancer cells in ascites mirror most of the molecular characteristics of cells in the primary tumor and at metastatic sites [32]. One explanation for this may be that the metastatic process in ovarian cancer is less complex than in most other types of cancer, since ovarian cancer cells metastasize mainly via ascites and not via the blood system [32]. Ascites contains single tumor cells and multicellular tissue-like aggregates, which have reduced proliferation and limited drug penetration resulting in decreased susceptibility to chemotherapy [33]. The composition of ascites has been reported to differ between untreated and treated patients since evolution of cancer stem cell-like cells occurs in response to platinum treatment [34].

We observed strong synergistic effects with APR-246 in combination with cisplatin in cancer cells from all tumor samples with *TP53* missense mutations, while additive or synergistic effects were observed in samples with wild type *TP53* or nonsense mutation resulting in truncated E346* p53. Notably, we observed synergy in cancer cells from both treated and untreated patients.

Further, we showed that APR-246 sensitizes primary ovarian cancer cells from a *TP53* mutant drug-resistant HGS patient to cisplatin. The cancer cells were isolated from ascites of a chemotherapy-treated patient carrying homozygous P278R mutant p53. APR-246 decreased the IC_50_ value of cisplatin in a dose-dependent manner, with a 3.6-fold decrease from 6.5 to 1.8 μM in the presence of 8 μM APR-246.

We also observed strong synergy with APR-246 in combination with the cisplatin analog carboplatin as well as with the anthracycline doxorubicin in the two samples tested, carrying homozygous *TP53* mutations (resulting in C135Y and P278R mutant p53, respectively). The main mechanisms of action of doxorubicin are inhibition of DNA and RNA synthesis by intercalation between base pairs of the DNA/RNA strands, and interference with the topoisomerase II enzyme, leading to double-strand breaks [35]. This triggers the DNA damage response and activation of the p53 pathway leading to apoptosis or senescence. Although it has been reported that inactivation of p53 can cause resistance to anthracyclines [36], the significance of p53 status for sensitivity to anthracyclines is less clear than its significance for sensitivity to platinum compounds.

In the present study, cancer cells from 7 of totally 10 patients had *TP53* missense mutations in the DNA binding region, two carried nonsense mutations, and one sample had wild type *TP53*. Two of the mutations have not previously been reported in ovarian cancer (L111Q and E346*). All 7 missense mutations are predicted to severely affect the function of p53 according to The p53 Web Site [6]. Most of them show transactivation activity below 15 % as compared to wild type p53 on 8 different p53-regulated promoters (*p21*, *MDM2*, *BAX*, *AIP*, *GADD45*, *Noxa*, *p53R2,* and *14-3-3-S*). Prediction of loss of activity based on phylogenetic conservation (SIFT) and other biochemical properties (Provean and Condel) indicates that all these mutations are deleterious. Notably, some recent studies indicate that patients with advanced ovarian/HGS cancer with various *TP53* mutations have different survival outcomes [37, 38].

All samples with cancer cells carrying missense mutations had medium or high levels of p53, while no p53 protein was detected in cells with wild type or nonsense mutations. High levels of p53 have been suggested to contribute to strong apoptosis-inducing effect of APR-246 [39]. Missense mutations in *TP53* are common in HGS ovarian cancer, but the disease is also characterized by the highest frequency of *TP53* nonsense or frameshift mutations in any cancer (15 % of *TP53* mutant tumors) [40].

## Conclusion

In the present study, we observed strong synergistic effect with APR-246 in combination with standard chemotherapy in primary cancer cells isolated from HGS cancer patients with various *TP53* missense mutations. Moreover, we show for the first time that APR-246, at clinically relevant concentrations, sensitizes primary ovarian cancer cells isolated from a drug-resistant *TP53* mutant patient to cisplatin. Drug-resistant HGS cancer patients have very poor prognosis since only palliative treatment is available. Our results suggest that combination treatment with APR-246 and DNA-damaging drugs has the potential to significantly improve the treatment of therapy-refractory HGS ovarian cancer.

### Ethics approval and consent to participate

The research project was approved by the local ethics committee at Karolinska Institutet (Dnr 2012/1933-31) and is in accordance with the principles of the Declaration of Helsinki. Written approved consent was obtained and the procedures were in agreement with Swedish law and ethical regulations.

### Consent for publication

Not applicable.

### Availability of data and material

The datasets on which the conclusions are based upon are presented in the article.

## Abbreviations

CI: Combination Index
FMCA: fluorometric microculture cytotoxicity assay
HGS cancer: High-Grade Serous cancer
MQ: 2-methylenequinuclidin-3-one
